# Ultrasound‐measured patellar tendon‐trochlear groove distance: A reliable and valid method with moderate correlation to computed tomography‐based knee lateralization parameters

**DOI:** 10.1002/jeo2.70322

**Published:** 2025-07-02

**Authors:** Naofumi Hashiguchi, Atsuo Nakamae, Akinori Nekomoto, Shunya Tsuji, Koji Takeda, Junya Tsukisaka, Kanji Goto, Nobuo Adachi

**Affiliations:** ^1^ Department of Orthopaedic Surgery, Graduate School of Biomedical and Health Sciences Hiroshima University Hospital Hiroshima Japan

**Keywords:** knee lateralization, Q‐angle – quadriceps angle, tibial tubercle‐posterior cruciate ligament, tibial tubercle‐trochlear groove, ultrasound, ultrasound‐measured patellar tendon‐trochlear groove

## Abstract

**Purpose:**

To evaluate the reliability and validity of ultrasound‐measured patellar tendon‐trochlear groove (USPT‐TG) distance as a novel method for assessing knee lateralization, and to determine its correlation with conventional computed tomography (CT)‐based measurements, including the tibial tubercle‐trochlear groove (TT‐TG), patellar tendon‐trochlear groove (PT‐TG) and tibial tubercle‐posterior cruciate ligament distances.

**Methods:**

This cross‐sectional study included patients who had undergone knee surgeries within the period from February 2023 to January 2024. The USPT‐TG measurements were performed using ultrasound imaging, 1 cm distal to the inferior patellar border. Inter‐rater and intra‐rater reliabilities were assessed using intra‐class correlation coefficients (ICCs). Correlations between the USPT‐TG and CT‐based parameters were analyzed using Pearson's correlation coefficient, and measurement agreement was evaluated using the Bland–Altman analysis.

**Results:**

A total of 75 patients (47 females and 28 males) with a mean age of 51.0 ± 23.5 years were included in this study. The USPT‐TG demonstrated excellent intra‐rater reliability (ICC: 0.98, 95% confidence interval [CI]: 0.97–0.99) and good inter‐rater reliability (ICC: 0.82, 95% CI: 0.75–0.88). The USPT‐TG had a strong correlation with the PT‐TG (r = 0.71, *p* < 0.0001) and a moderate correlation with the TT‐TG (*r* = 0.42, *p* = 0.0002). The mean measurements were: USPT‐TG, 8.3 ± 2.8 mm; TT‐TG, 12.5 ± 3.5 mm and PT‐TG, 9.0 ± 2.9 mm. The Bland–Altman analysis showed acceptable agreement between the inter‐rater reliability of the USPT‐TG distance (bias: 0.59 ± 2.21 mm, 95% limits of agreement [LOA]: −3.73 to 4.92 mm), USPT‐TG versus TT‐TG measurements (bias: 4.20 ± 3.47 mm, 95% LOA: −2.61 to 11.01 mm) and USPT‐TG versus PT‐TG measurements (bias: −0.71 ± 2.17 mm, 95% LOA: −4.96 to 3.54 mm).

**Conclusion:**

The USPT‐TG is a reliable and valid method for assessing knee lateralization and demonstrates a moderate‐to‐strong correlation with the TT‐TG and PT‐TG measurements. This radiation‐free technique may offer a practical alternative to traditional imaging methods, particularly in settings where radiation exposure should be minimized or access to advanced imaging is limited. Further research is needed to establish the diagnostic thresholds and validate this technique in patients with patellar instability.

**Levels of Evidence:**

Level II.

**Abbreviations**
ACL: anterior cruciate ligament
CI: confidence interval
CT: computed tomography
FTA: femorotibial angle
ICC: intra‐class correlation coefficient
LOA: limits of agreement
MRI: magnetic resonance imaging
PT‐TG: patellar tendon‐trochlear groove
TT‐PCL: tibial tubercle‐posterior cruciate ligament
TT‐TG: tibial tubercle‐trochlear groove
US: ultrasound
USPT‐TG: ultrasound‐measured patellar tendon‐trochlear groove

## INTRODUCTION

The accurate assessment of knee lateralization is crucial for the diagnosis and management of patellofemoral disorders [25]. Traditional imaging modalities, particularly computed tomography (CT), have been widely used for knee lateralization, with the tibial tubercle‐trochlear groove (TT‐TG) distance being a reliable measure [18]. However, conventional methods have limitations, including radiation exposure risks [19] and restricted accessibility in smaller medical facilities, highlighting the need for more accessible and cost‐effective assessment methods.

Ultrasound (US) imaging has shown promise in evaluating patellofemoral joint conditions [4, 21]. While direct measurement of TT‐TG via US presents technical challenges, we explored alternative parameters, particularly the patellar tendon‐trochlear groove (PT‐TG) distance. Recent magnetic resonance imaging (MRI)‐based studies have validated PT‐TG measurements, demonstrating strong correlations with traditional TT‐TG measurements [14]. Additionally, US offers advantages such as real‐time imaging, cost‐effectiveness and absence of radiation exposure [8]. Recent studies have demonstrated the potential of ultrasound in evaluating patellofemoral alignment, though its reliability and correlation with established CT measurements require further investigation.

The development of reliable ultrasound‐based measurement techniques could significantly improve the clinical assessment of patellofemoral disorders [11]. Current clinical practice relies heavily on static imaging, but the dynamic nature of patellofemoral instability suggests that real‐time assessment methods could provide valuable additional information. However, there is currently no standardized ultrasound method for measuring knee lateralization parameters that has been thoroughly validated against CT measurements. Furthermore, the relationship between ultrasound‐based measurements and traditional CT parameters remains unclear, limiting the clinical application of this promising technique.

This study aimed to comprehensively evaluate the relationship between US‐based knee lateralization assessment methods and conventional CT‐based measurements.

We hypothesized that USPT‐TG would demonstrate diagnostic accuracy comparable to established knee lateralization indices while providing additional dynamic assessment capabilities not available with conventional CT imaging.

## METHODS

### Study design and participants

This cross‐sectional study was conducted at a single institution between February 2023 and January 2024. The study was approved by the institutional review board of our institution (approval number: E2024‐0008) and adhered to the STARD statement guidelines for diagnostic studies.

This study included consecutive patients who underwent joint replacement, osteotomy for osteoarthritis, arthroscopy for meniscal injury or anterior cruciate ligament (ACL) reconstruction for ACL injury. Patient enrolment was conducted in a consecutive manner, and all eligible patients who presented during the study period were invited to participate. No randomization was performed. The exclusion criteria were knee flexion contracture exceeding 30° (to ensure reliable knee lateralization measurements), history of previous knee trauma unrelated to the current surgical indication, lower‐limb paralysis, history of patellar instability and patients who declined to provide informed consent for the study procedures.

### Study objectives

The specific objectives of this study were threefold: first, to establish the inter‐rater reliability of USPT‐TG measurements; second, to determine the correlation between USPT‐TG and CT‐based parameters; and third, to assess the agreement between these measurement methods in the clinical setting.

### Measurement technique of the USPT‐TG distance

**Figure 1:**
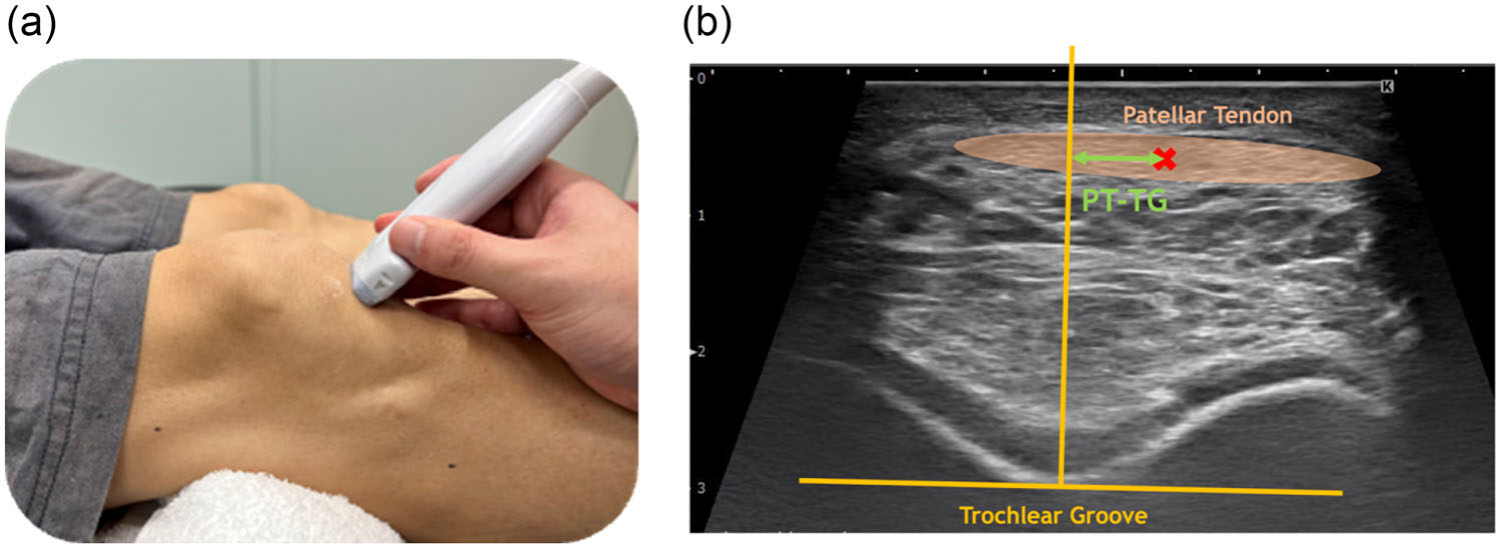
(a) Position of the transducer head in supine position; (b) ultrasound image of the patellar tendon and trochlear groove (PT‐TG).

The US machine used was the SONIMAGE HS2 (KONICA MINOLTA), and the transducer model used was the L11‐3. The measurement method was confirmed by checking whether the preoperative CT was performed in an extended or slightly flexed position. Based on the CT angle, the transducer was positioned 1 cm distal to the inferior edge of the patella in a short‐axis orientation (Figure 1a). US examinations were performed by an experienced orthopaedic surgeon (N.H.) with >10 years of experience in musculoskeletal US. Images of the patellar tendon and trochlear groove of the femur were captured [11] and imported into ImageJ (National Institutes of Health) to perform the measurements. Before measurements, spatial calibration was performed for each image using the following protocol: (1) The 1‐cm scale bar displayed on each ultrasound image was used as a reference for calibration. (2) Using the straight line selection tool, a line was drawn along the 10 mm scale bar. (3) The Set Scale function was used to calibrate the image by entering the known distance (10 mm) and setting the unit of measurement. (4) The Global option was selected to maintain consistent calibration across all measurements. The USPT‐TG was measured as the vertical distance between the centre of the patellar tendon and the lowest point of the trochlear groove where the tangents were drawn (Figure 1b). The USPT‐TG measurements were performed independently by two orthopaedic surgeons (N.H. and K.G.) to assess inter‐rater reliability. For the intra‐rater reliability assessment, one surgeon (K.G.), who was blinded to the study objectives, performed repeated measurements at two separate time points.

### Measurement of other factors

The TT‐TG, PT‐TG and tibial tubercle‐posterior cruciate ligament (TT‐PCL) distances were measured on CT images using the PACS system by an experienced orthopaedic surgeon (N.H.) with >10 years of clinical experience. These parameters are widely used to evaluate tibial lateralization and serve as primary comparative measurements for USPT‐TG analysis. The TT‐TG distance measurement followed the technique according to the previous studies [20, 24]. While the PT‐TG measurements were based on a modification of the MRI‐based approach of Hinckel [14], which originally measured the patellar tendon at the tibial tubercle level. To maintain consistency with our US imaging, we measured the PT‐TG distance on axial CT images 1 cm distal to the inferior patellar border, between the centre of the patellar tendon and the trochlear groove point used in conventional TT‐TG measurements.

The TT‐PCL measurements followed a previous study by Anley et al., which utilized bony landmarks while excluding cartilaginous references [2]. The trochlear groove was identified as the deepest point where the posterior femoral condylar cortex was clearly defined. The posterior condylar line was tangential to the posterior condylar cortex, and a trochlear line was drawn perpendicular to the posterior tibial line and passed through the deepest point of the trochlear groove [9, 20]. The tibial tubercle position was marked at the most proximal image level, where the patellar tendon made complete contact with the tibial tubercle, and the tubercle was clearly marked at the centre of the tendon. Additional radiographic parameters were assessed using the weight‐bearing full‐length lower extremities and anteroposterior radiographs of the knee. As described by Smith et al., the *Q*‐angle served as an established indicator of the patellar tendon extensor mechanism [22], whereas the femorotibial angle (FTA) provided sensitive detection of knee varus/valgus alignment [6, 12]. The Kellgren–Lawrence classification was used to grade the severity of osteoarthritic changes [1, 15].

### Sample‐size calculation and statistical analysis

Based on a previous clinical study by Camp where TT‐TG measurements between CT (mean, 16.9 mm; range, 8.3–25.8 mm) and MRI (mean, 14.7 mm; range, 1.5–25.1 mm) were compared in 59 knees with patellar instability, we estimated that more than 59 cases would be necessary for our comparison [7]. Anticipating that the USPT‐TG values would be lower than the TT‐TG measurements with moderate correlation, we determined that 75 specimens would be required using a two‐tailed type 1 error rate of 5%, power of 0.80, and *r* ^2^ of 0.1. Sample size calculations were performed using G*Power Version 3.1.9.6 [10]. All measurements were expressed as percentages or means with standard deviations. Inter‐rater and intra‐rater reliabilities for USPT‐TG measurements were evaluated using the R software. Intra‐rater reliability was assessed using intra‐class correlation coefficients (ICCs; 1, 2) with a one‐way random‐effects model for consistency, whereas inter‐rater reliability was calculated using ICC (2, 1) with a two‐way random‐effects model for absolute agreement [16]. An ICC of >0.75, 0.75–0.40 and <0.40 was considered excellent, fair and poor, respectively [17]. The inter‐rater reliabilities for USPT‐TG were assessed using the Bland–Altman 95% limit of agreement (LOA) in the GraphPad Prism 10 [5]. Pearson's correlation coefficient was used to assess the association between USPT‐TG, CT‐based measurements (TT‐TG, PT‐TG and TT‐PCL), and radiographic parameters (*Q*‐angle and FTA). Statistical significance was set at *p* < 0.05.

## RESULTS

A total of 75 patients (47 females and 28 males) were included in this study. The demographic characteristics revealed a mean age of 51.0 ± 23.5 years (range: 12–95), mean height of 1.59 ± 0.10 m (range: 1.36–1.78), mean weight of 64.7 ± 13.5 kg (range: 36.9–96.4) and mean body mass index of 25.5 ± 4.8 kg/m^2^ (range: 16.8–40.8).

The study population comprised 23 cases of arthroplasty (30.7%), 15 cases of osteotomy (20.0%), 2 cases of arthroscopy (2.7%) and 35 cases of ACL reconstruction (46.7%). The distribution of Kellgren‐Lawrence classification showed 25 patients with Grade 0 (33.3%), 10 with Grade 1 (13.3%), 12 with Grade 2 (16.0%), 7 with Grade 3 (9.3%) and 21 with Grade 4 (28.0%) disease.

**Figure 2:**
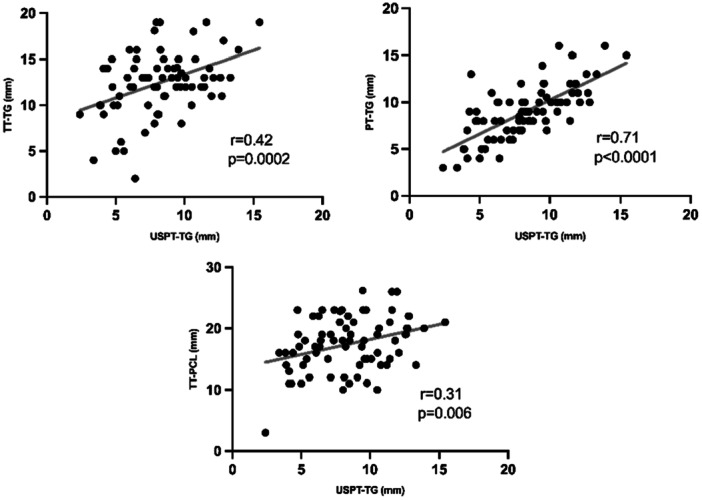
Pearson's correlation between the USPT‐TG and CT‐based parameters. CT, computed tomography; USPT‐TG, ultrasound‐measured patellar tendon‐trochlear groove.

The radiographic parameters revealed a mean *Q*‐angle of 7.8 ± 5.3° (range: −6.7 to 24.5) and a mean FTA of 177.9 ± 7.7° (range: 162.7–201.8). The knee lateralization measurements showed mean values of USPT‐TG at 8.3 ± 2.8 mm (range: 2.4–15.4), TT‐TG at 12.5 ± 3.5 mm (range: 2.0–19.0), PT‐TG at 9.0 ± 2.9 mm (range: 3.0–16.0) and TT‐PCL at 17.4 ± 4.4 mm (range: 3.0–26.2). The reliability analysis demonstrated excellent metrics, with intra‐rater and inter‐rater ICC for USPT‐TG measurements (Table 1). The correlation analysis revealed significant associations between USPT‐TG and CT‐based parameters, with the strongest correlation observed with PT‐TG (*r* = 0.71, *p* < 0.0001), followed by moderate correlation with TT‐TG (*r* = 0.42, *p* = 0.0002), and weak correlation with TT‐PCL (*r* = 0.31, *p* = 0.006) (Figure 2). No significant correlations were found between USPT‐TG and radiographic parameters (*Q*‐angle: *r* = 0.016, *p* = 0.89; FTA: *r* = 0.17, *p* = 0.14).

**Table 1:** Inter‐ and intra‐rater reliability for USPT‐TG.

Results of interclass reliability calculations		
Interclass reliability	ICC	95% CI
Inter‐rater reliability	0.82	0.75–0.88
Intra‐rater reliability	0.98	0.97–0.99

Abbreviations: CI, confidence interval; ICC, interclass correlation coefficient; USPT‐TG, ultrasound‐measured patellar tendon‐trochlear groove.

**Figure 3:**
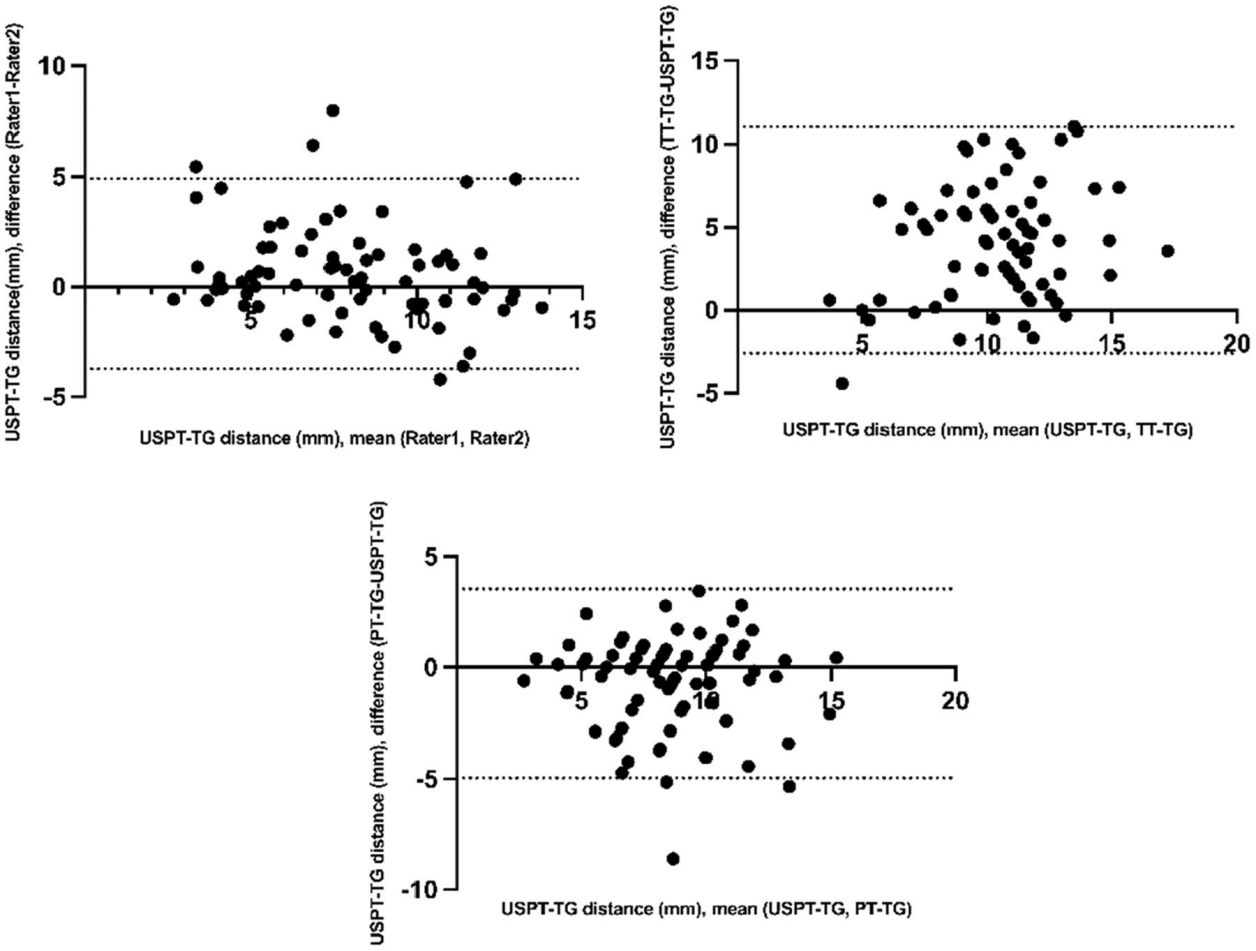
The Bland–Altman analysis for the USPT‐TG, TT‐TG and PT‐TG. PT‐TG, patellar tendon‐trochlear groove; TT‐TG, tibial tubercle‐trochlear groove; USPT‐TG, ultrasound‐measured patellar tendon‐trochlear groove.

The Bland–Altman analysis showed acceptable agreement between observers for USPT‐TG measurements and demonstrated good interchangeability with PT‐TG measurements (Table 2 and Figure 3).

**Table 2:** The results of the Bland–Altman analysis for the USPT‐TG, TT‐TG and PT‐TG.

Results of Bland‐Altman analysis		
Bland–Altman analysis	Difference bias ± SD, mm	95% LOA, mm
USPT‐TG of Rater 1 vs. USPT‐TG of Rater 2	0.59 ± 2.21	−3.73 to 4.92
USPT‐TG vs. TT‐TG	4.20 ± 3.47	−2.61 to 11.01
USPT‐TG vs. PT‐TG	−0.71 ± 2.17	−4.96 to 3.54

Abbreviations: LOA, limits of agreement; PT‐TG, patellar tendon‐trochlear groove; SD, standard deviation; TT‐TG, tibial tubercle‐trochlear groove; USPT‐TG, ultrasound‐measured patellar tendon‐trochlear groove.

## DISCUSSION

The USPT‐TG demonstrated excellent reliability metrics (intra‐rater ICC: 0.98 and inter‐rater ICC: 0.82) and showed varying degrees of correlation with established CT‐based measurements. Most notably, USPT‐TG demonstrated a strong correlation with PT‐TG (*r* = 0.71, *p* < 0.0001) but only a moderate correlation with TT‐TG (*r* = 0.42, *p* = 0.0002). These findings suggest that while USPT‐TG may serve as a reliable measurement tool, its relationship with traditional TT‐TG measurements is more modest than initially anticipated. The stronger correlation with PT‐TG is particularly noteworthy and may be explained by the anatomical similarity of the measurement points, as both methods measure from the patellar tendon rather than the tibial tubercle. These reliability values align favourably with previously reported reliability measures for conventional methods [3, 13, 26].

The observed correlations between USPT‐TG and conventional measurements (*r* = 0.42 for TT‐TG, *r* = 0.71 for PT‐TG and *r* = 0.31 for TT‐PCL) were similar to those reported in previous comparative studies on knee lateralization parameters. A systematic review of existing measurement techniques has shown that TT‐TG and TT‐PCL measurements typically demonstrate correlation coefficients ranging from 0.48 to 0.79 in normal knees [9, 26]. Studies comparing different imaging modalities have reported correlation coefficients between CT‐ and MRI‐based TT‐TG measurements ranging from 0.53 to 0.57. These findings suggest that USPT‐TG measurements may provide correlation strengths comparable to those observed for other established lateralization parameters.

Despite the findings, this study had some limitations that warrant consideration. First, our study population primarily consisted of patients with osteoarthritis, meniscal injuries, and ACL injuries; hence, cases of patellar instability, which is the primary clinical condition where TT‐TG measurements are traditionally utilized, were lacking. This limitation affects the generalizability of our findings to patients with patellar instability. Second, while CT‐based measurements provide consistent imaging regardless of operator experience, US imaging quality can vary significantly depending on the expertise of the sonographer [23]. Although our study demonstrated high reliability among experienced operators, other physicians may not be able to obtain similar images because imaging was performed by an experienced physician. Therefore, the learning curve and technical expertise required for accurate USPT‐TG measurement in general clinical practice remain to be determined. Third, while our results support the reliability of USPT‐TG measurements, the diagnostic threshold values and therapeutic decision‐making criteria are yet to be established. While this study establishes USPT‐TG's technical reliability, its diagnostic performance in patellar instability requires dedicated investigation. Future multicenter collaborations will be critical to validate cutoff values in instability populations.

The findings of this study support the potential clinical utility of USPT‐TG as a knee lateralization assessment tool. Our results demonstrate that USPT‐TG provides reliable measurements and shows varying degrees of correlation with established CT‐based parameters, specifically a strong correlation with PT‐TG (*r* = 0.71) but only a moderate correlation with TT‐TG (*r* = 0.42). The advantages of US imaging include its non‐invasive nature, absence of radiation exposure and real‐time visualization capability, which makes USPT‐TG particularly attractive for clinical practice. Although additional research is needed to establish diagnostic thresholds and validate their use in patients with patellar instability, USPT‐TG shows promise as a practical alternative to conventional imaging methods, especially in settings where radiation exposure should be minimized or where access to advanced imaging is limited.

## CONCLUSION

While USPT‐TG showed promising reliability metrics and associations with conventional measurements, further validation in patients with patellar instability is required before its widespread clinical implementation. This method may serve as a practical alternative to conventional imaging, particularly in settings where radiation exposure must be minimized and access to imaging modalities is limited.

## AUTHOR CONTRIBUTIONS

**Naofumi Hashiguchi**: Writing; conceptualization; methodology; investigation; data collection; editing. **Atsuo Nakamae**: Methodology; investigation; review; editing. **Akinori Nekomoto**: Data collection. **Shunya Tsuji**: Data collection. **Koji Takeda**: Data collection. **Junya Tsukisaka**: Data collection. **Kanji Goto**: Data collection; validation. **Nobuo Adachi**: Review.

## CONFLICT OF INTEREST STATEMENT

The authors declare no conflicts of interest.

## ETHICS STATEMENT

The study was approved by the institutional review board of our institution (approval number: E2024‐0008) and adhered to the STARD statement guidelines for diagnostic studies. This study was conducted with an opt‐out approach. Information about the study was publicly disclosed on our institution's website, providing patients with the opportunity to opt out.

## Supporting information

Supporting Information.

## Data Availability

The data that support the findings of this study are available from the corresponding author upon reasonable request.
